# Supplementary figures and images for: Activation of the P2RX7/IL-18 pathway in immune cells attenuates lung fibrosis
Source: eLife. 2024 Feb 1;12:RP88138. doi: 10.7554/eLife.88138 (PMC10945561; doi:10.7554/eLife.88138)

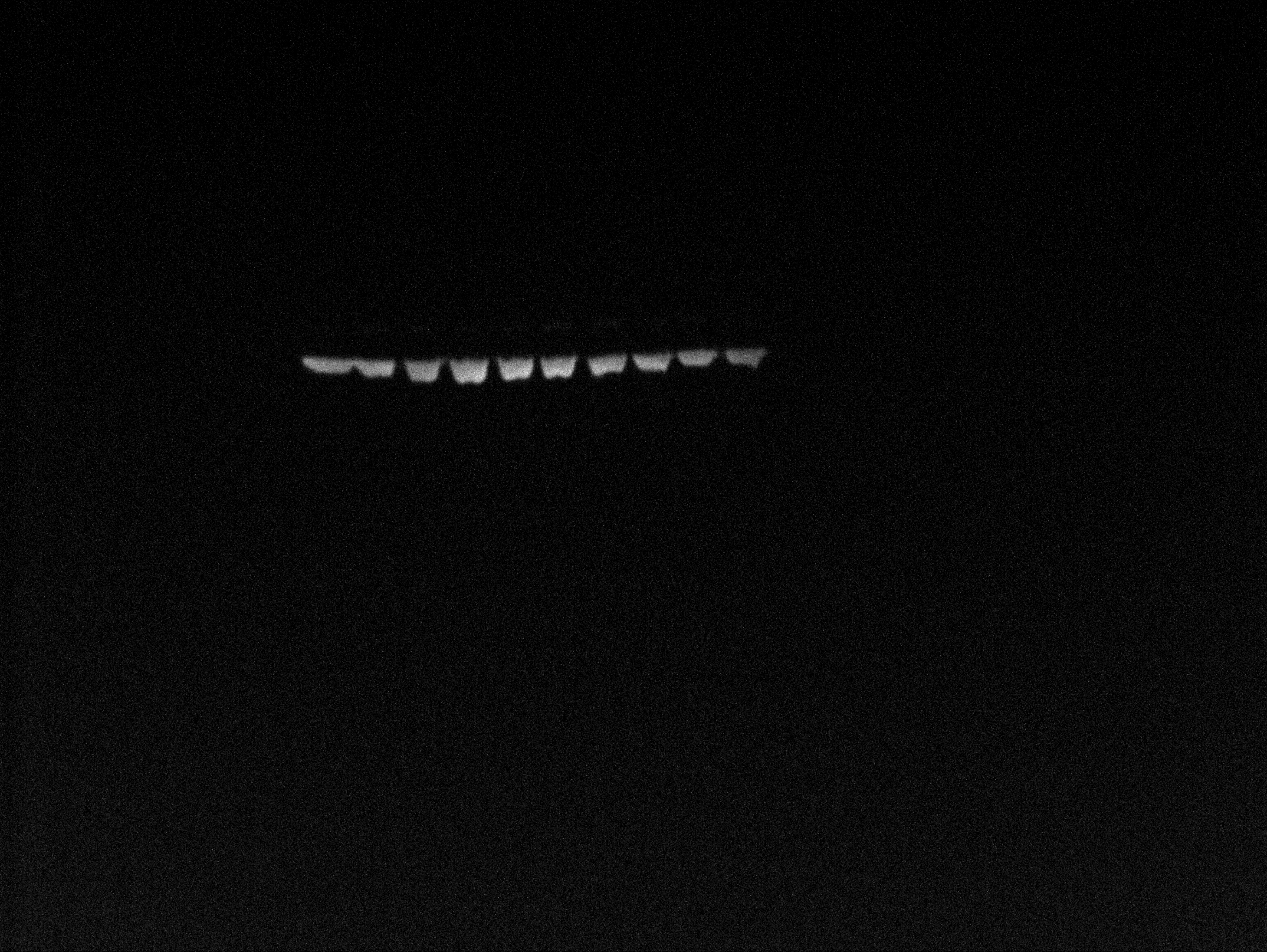

Supplement: Figure 4—figure supplement 2—source data 2. [file elife-88138-fig4-figsupp2-data2.zip › raw data WB tiff/ACTB WB.tif]

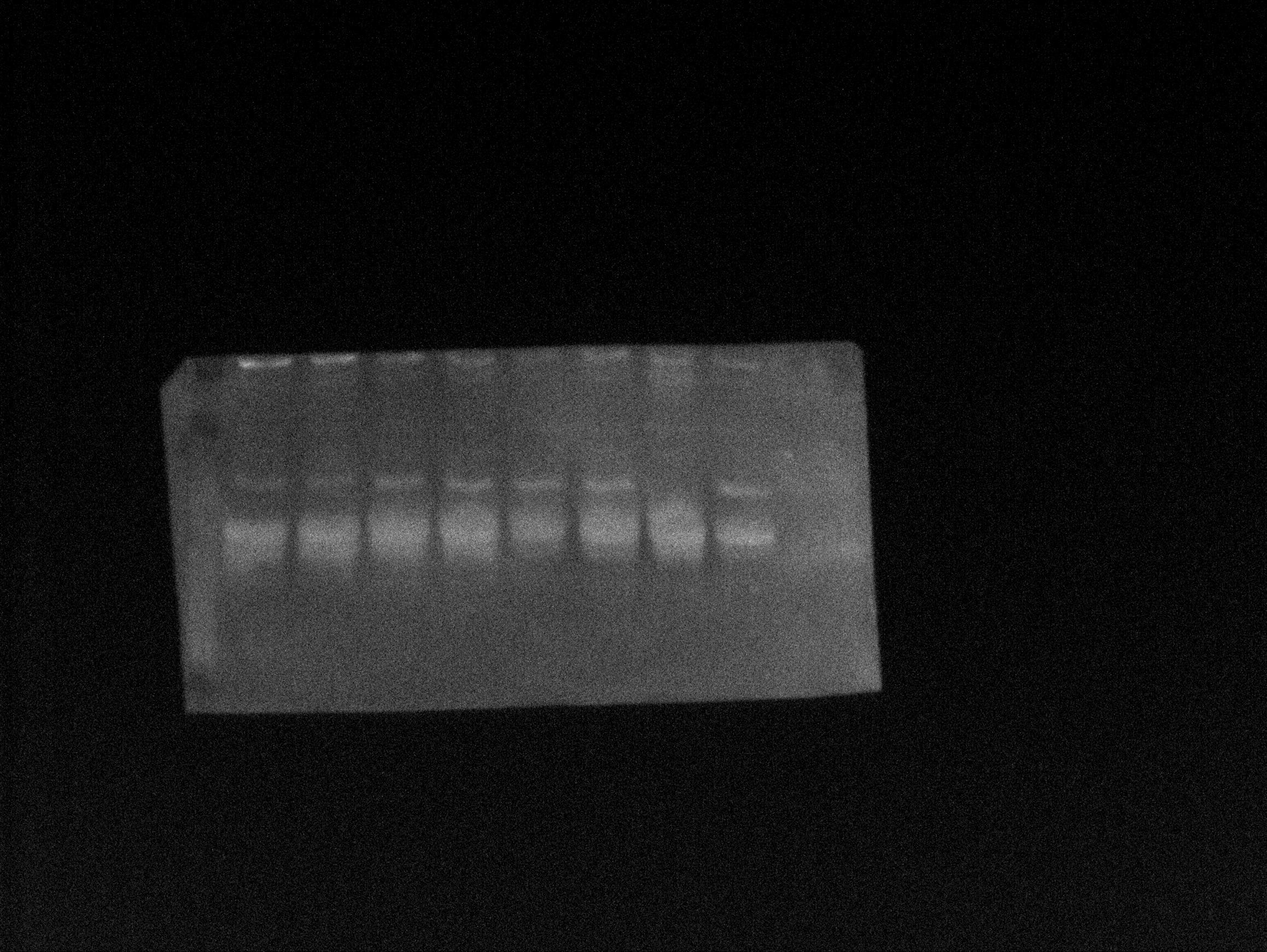

Supplement: Figure 4—figure supplement 2—source data 2. [file elife-88138-fig4-figsupp2-data2.zip › raw data WB tiff/IL18 WB.tif]

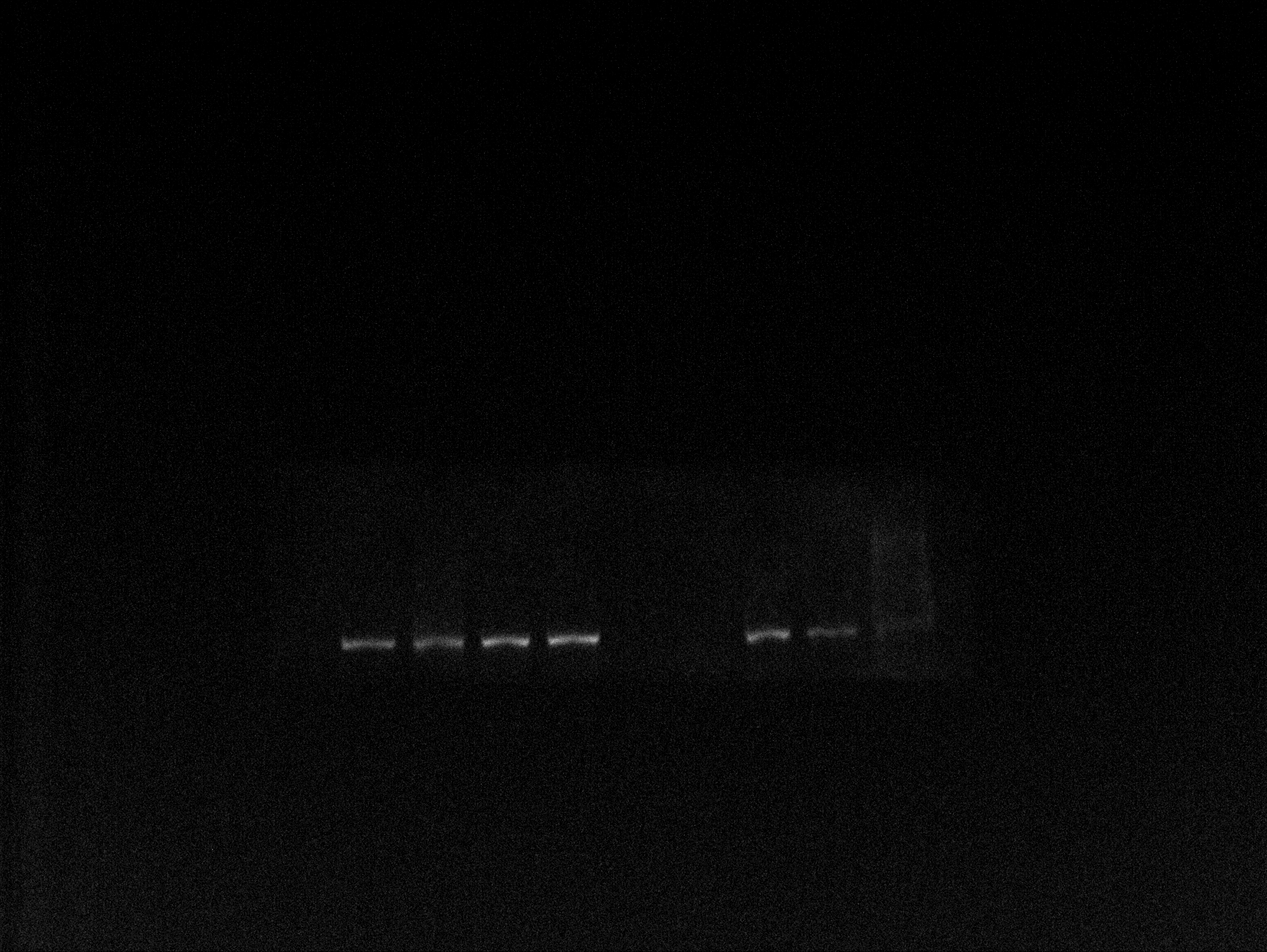

Supplement: Figure 4—figure supplement 2—source data 2. [file elife-88138-fig4-figsupp2-data2.zip › raw data WB tiff/NLRP3 WB.tif]

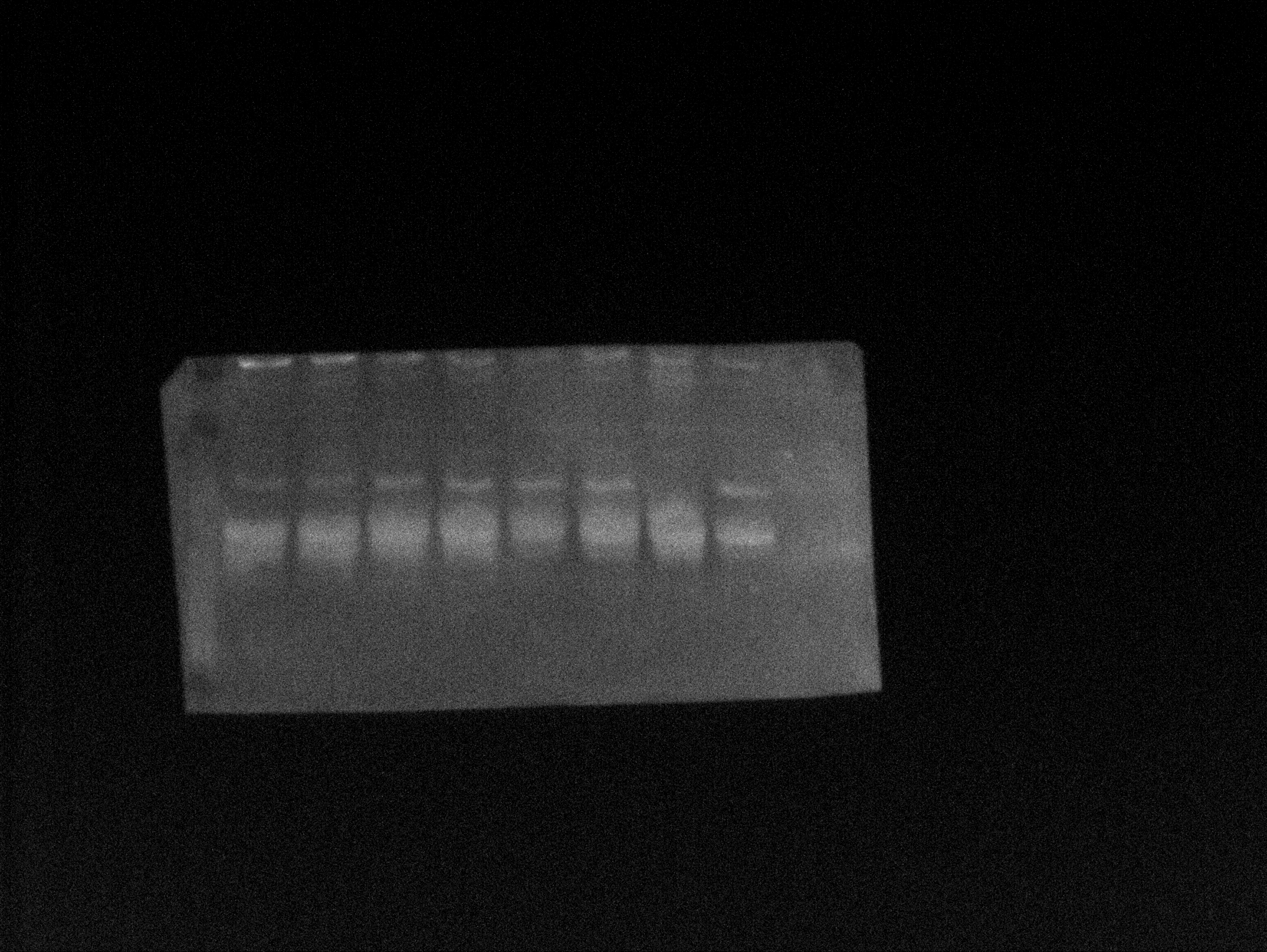

Supplement: Figure 4—figure supplement 2—source data 3. [file elife-88138-fig4-figsupp2-data3.zip › Figure 4-figure supplement 2-source data 3.tif]

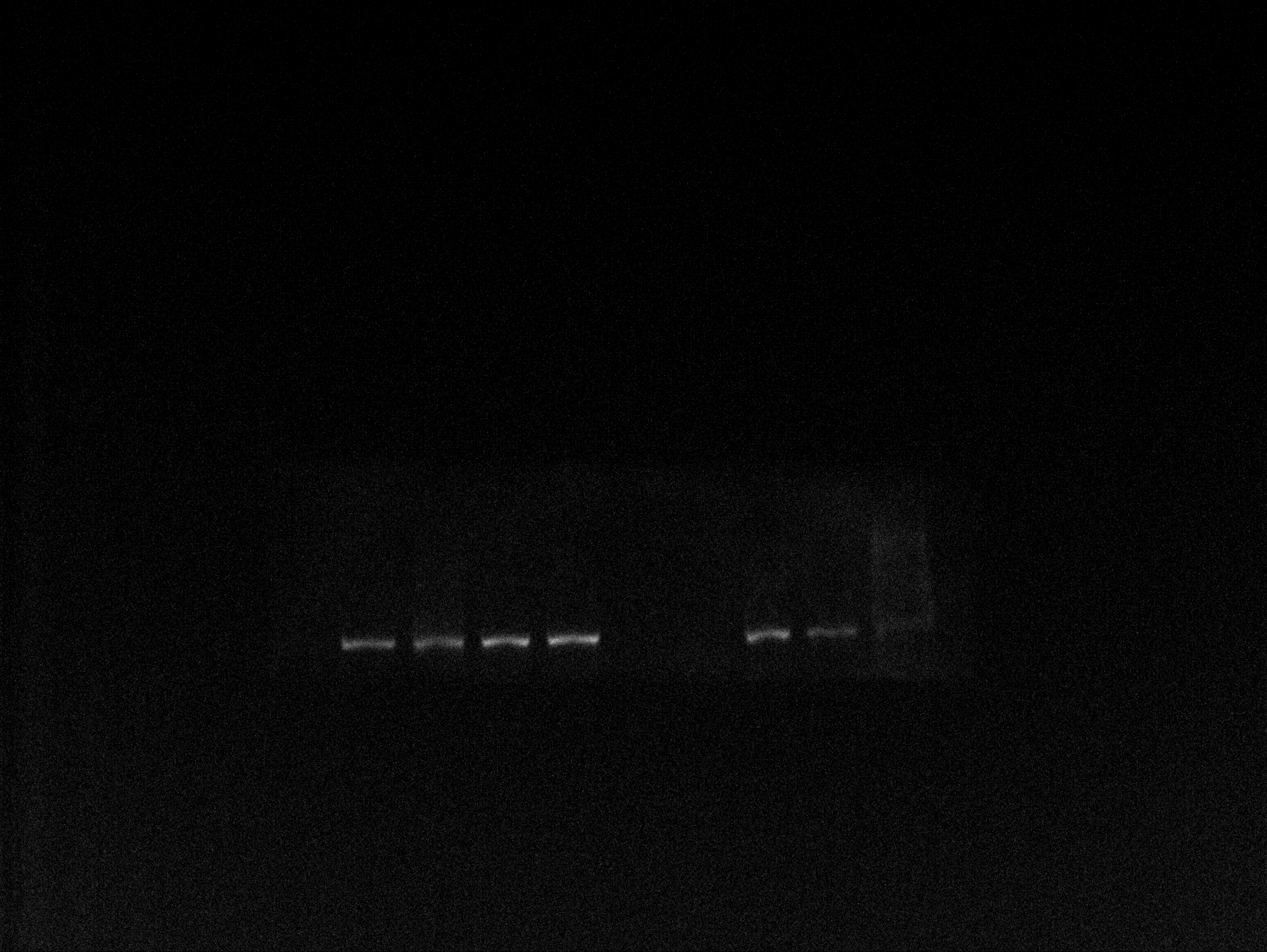

Supplement: Figure 4—figure supplement 2—source data 4. [file elife-88138-fig4-figsupp2-data4.zip › Figure 4-figure supplement 2-source data 4.tif]

Panel 4F

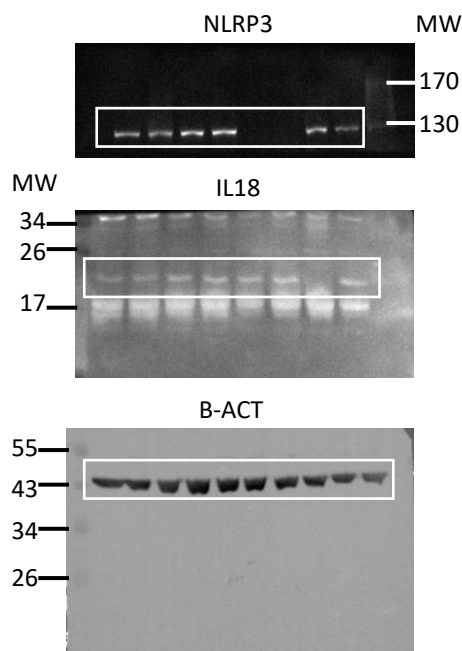

F

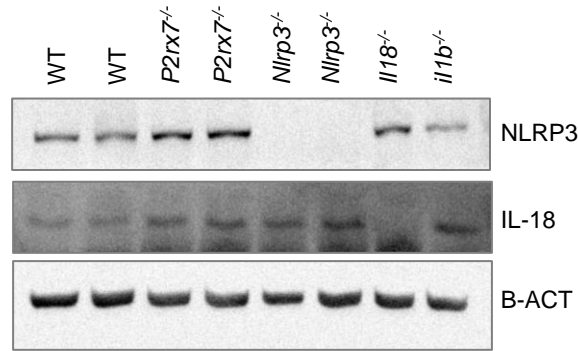

Supplement: Figure 4—figure supplement 2—source data 5. [file elife-88138-fig4-figsupp2-data5.pdf]
